# Conscious Sedation in Dentistry

**DOI:** 10.3390/medicina55120778

**Published:** 2019-12-07

**Authors:** Luca Fiorillo

**Affiliations:** Department of Biomedical and Dental Sciences, Morphological and Functional Images, University of Messina, Policlinico G. Martino, Via Consolare Valeria, 98100 Me, Italy; lfiorillo@live.it

**Keywords:** oral health, general health, well-being, sedation, interdisciplinary, fear, phobia

## Abstract

Invasive dental procedures can be performed only with local anesthesia; in some cases, it may be useful to combine the administration of drugs to obtain anxiolysis with local anesthesia. Sedation required level should be individually adjusted to achieve a proper balance between the needs of the patient, the operator, and the safety of the procedure. Surgical time is an important factor for post-operative phases, and this could be greatly increased by whether the patient interrupts the surgeon or if it is not collaborative. In this manuscript some dentistry-used methods to practice conscious sedation have been evaluated. This manuscript could be a useful reading on the current state of conscious sedation in dentistry and an important starting point for future perspectives. Surely the search for safer drugs for our patients could have beneficial effects for them and for the clinicians.

Difficult situations and patients often present themselves to the dentist specialist, whose treatment requires special methods. Frequently, the emotional and anxious component makes the treatment of some subjects particularly difficult [1]. The use of conscious sedation proved to be effective in winning and controlling these phenomena of aversion for the dentist. This practice—now widely used and well defined in specific protocols—allows, together with other appropriate measures, results in excellent satisfaction in many clinical cases. This method, as well as being effective, has the great advantage of being safe and of routine use in an ambulatory environment [2,3,4].

The methods of administration and the sedative drugs could be different. Moreover, it is necessary to at least mention that some schools of thought practice non-pharmacological techniques such as hypnosis [5,6].

During sedation, the reduction or abolition of physiological and psychological responses of the patient to surgery are obtained, without, however, a loss of consciousness, collaboration, and protective reflexes; it is used to treat moderately anxious patients and allows for a calm and relaxed patient during therapy, with anterograde amnesia. Sedation can be performed by different methods orally or parenterally with benzodiazepines or inhaled with nitrous oxide. Sedation with benzodiazepines is not recommended under the age of 16 and in children in whom nitrous oxide (conscious sedation) is preferable. Some benzodiazepines administered orally produce sedation similar to intravenous techniques; temazepam has a short half-life (8 h) and is preferable to diazepam (half-life of 20–30 h). Intravenous sedation has immediate action; requires skill and experience in intravenous and drug administration; for this reason, it is contraindicated for inexperienced operators. Intramuscular absorption is slower and inconstant [4]. Midazolam is two times powerful than diazepam, has a shorter half-life of 2 h, rapid onset and recovery with anterograde amnesia. It should however be reiterated that the sedation technique requires special precautions:

- Guarantee a recovery time and postoperative medical surveillance in the dental office of at least 1 h.
- Discharge the accompanied patient.
- It requires to have a specific benzodiazepine antagonist (flumazenil) in the dental office for overdose emergencies management.
- Warn the patient to avoid driving or taking on responsibilities for the next 12–24 h [4,5].

Nitrous oxide is a colorless gas with a sweetish taste. It is an effective analgesic/anxiolytic agent that causes depression and euphoria in the central nervous system (CNS) with negligible effects on the respiratory and cardiovascular system. The analgesic effect of nitrous oxide is manifested by the release of endogenous opioid peptides with the consequent activation of opioid receptors and descending receptors of Gamma-aminobutyric acid type A (GABA-A) and the noradrenergic metabolic sequence which modifies the spinal nociceptive process. The anxiolytic effect involves the activation of GABA receptors through the benzodiazepine binding sites. Nitrous oxide, in the blood, is 34 times soluble than nitrogen. It is quickly eliminated and 98% through the lungs. When it is eliminated from the body, for physical reasons, it could cause a desaturation of O_2_. To avoid the inconvenience, it is important to give patients 100% oxygen for a period of 3–5 min. Nitrous oxide causes a minor depression in the heart flow while peripheral resistance slightly increases; because of this the blood pressure remains unchanged. This gas causes a minimum weakening of the protective reflexes, such as coughing and swallowing, in the patient despite having liquids in his mouth. There is no danger because the patient could take advantage of these fundamental reflexes. It is obvious that if the patient loses consciousness these reflexes could be compromised. It is necessary the patient always remains conscious during sedation. For some patients, especially those suffering from severe anxiety, the feeling of loss of control could be a problem and claustrophobic patient could find the imprisoning nasal mask and have the feeling of not breathing well. Inhalation sedation with N_2_O/O_2_ is based on the use of an inhalation mixture of nitrous oxide and oxygen administered in different percentages. Today, as an alternative to deep sedation and narcosis, it could be used on an outpatient basis for children who should undergo modest procedures, with rectal diazepam. This consists of rectal administration of a short-lived benzodiazepine [4,5,6,7,8,9,10].

Diazepam is supplied in the form of 5 mg/2.5 mL micro-cells or 10 mg/2.5 mL to be used based on the age and weight of the pediatric patient. The achievement of different sedation conditions should be carefully evaluated also based on the requests made by the oral surgeon, and the general health situations of our patient [11,12,13]. Surely the time of sedation is one of the most important factors to evaluate, depending on the presence of a first-level surgery or an elective surgery such as the implant surgery [14,15,16,17,18,19,20,21].

The use of these systems is strongly recommended to subjects, adults and children, intolerant to dental care by supporting them in equipped and adequately organized healthcare facilities to safely apply these techniques [22,23,24,25].
